# Broken Promises – The Probable Futurity of the Laboring Class (Re-Assessed)

**DOI:** 10.1007/s41463-022-00128-2

**Published:** 2022-06-28

**Authors:** Michael S. Aßländer

**Affiliations:** International Institute Zittau, Technical University Dresden, Markt 23, 02763 Zittau, Dresden, Germany

**Keywords:** Political economy, Work relationship, Gig economy, Globalization, Social security

## Abstract

Over the past two decades, work relations have changed dramatically. New phenomena like “gig-economy” or “crowd work” not only constitute precarious working conditions but also contradict with our social esteem of work resulting from the social theories of the classical economy of the eighteenth and nineteenth centuries. The central focus of classical economists on building an educated and disciplined workforce provided not only the base for the upcoming industrial society but also resulted in a work-based society where “being employed” became the precondition for social security and social participation. It is the aim of this contribution to show how our positive attitudes towards work, established by the political economic theories of the eighteenth and nineteenth centuries, are jeopardized by the social changes in post-industrialized societies, due to the effects of globalized economies, digitalization and changed industrial relations. This has also far-reaching consequences for managerial theories based on conceptions like meaningful work or discussions about social responsibilities vis-à-vis employees as primary stakeholder groups.

## Introduction

Over the last decades, numerous management theories have recognized the importance of work satisfaction as a key factor for increasing productivity, raising work performance and fostering loyalty of the workforce (Albrecht et al. 2021). While in the first phase of industrialization, the organization of work mainly was seen as a technical task (e.g. Taylor 2019 [1911]; Mayo 1998 [1933]), contemporary management theories focus also on psychological, motivational and moral aspects of labor relations. Thus, for instance, labor relations have become a prominent topic in the contemporary CSR-debate and are discussed from various angles (e.g. Valentine and Fleischman 2008; Shuili et al. 2015). Seen from stakeholder theory, employees are defined as primary stakeholders of corporations, which implies specific duties of corporations vis-à-vis their employees (e.g. Ferrary 2008; Kaler 2009). Especially, over the last years the topic of meaningful work has gained broad attention in management as well as in business ethics literature. In this vein, various dimensions of “meaningful work” have been discussed, like the interrelation of leadership and job satisfaction (e.g. Arnold et al. 2007; Lips-Wiersma et al. 2020), spirituality and its influence on the meaning of work (e.g. Lips-Wiersma 2002; Sheep 2006), or meaningfulness as a psychological precondition for job satisfaction (e.g. May et al. 2004). Several empirical studies have analyzed generation differences when assessing the meaningfulness of work (e.g. Weeks and Schaffert 2019) or key factors and constraints that influence work satisfaction. Other empirical studies have tried to evidence positive correlations between meaningfulness and its effects on loyalty, job satisfaction or workers’ motivation. In addition, several theoretical contributions have endeavored to give normative foundations for employees’ duties vis-à-vis their employees and, in this vein, have referred to ethical conceptions, like virtue ethics (Beadle and Knight 2012), Kantian philosophy (e.g. Bowie 1998), or Catholic Social Teaching (e.g. Tablan 2015).

Despite all differences of the various approaches, their different underlying theories and their different methodological procedures, all conceptions refer to a typical work-relation based on an employer at the one side, hiring employees on a long-term base, on the other. Seen from this, often instrumental, perspective, good work-relations benefit both, employers and employees. While employers profit from an increased loyalty of their employees, an enhanced work-performance and productivity and an increase of motivation, employees benefit from increased autonomy, the possibility of self-realization and identification with one’s work.

However, such idea of employer-employee relation mirrors an outdated ideal of an industrial society, which today becomes questionable due to the effects of globalized economies, digitalization and changed industrial relations. Over the past two decades, work relations have changed dramatically. New phenomena like “gig-economy” or “crowd work” not only constitute precarious working conditions but also contradict with our social esteem of work resulting from the social theories of the classical economy of the eighteenth and nineteenth centuries. In this new post-industrial society, regular employment on long-term conditions more and more becomes an exception. Management conceptions like meaningful work, ideas of workers’ participation rights, but also social security systems or work-related patterns of social identification and belongings based on this old understanding of life-long employment and clear career pathways become brittle in the new economy. New developments in the labor-market, like outsourcing jobs to cheap “telemigrants”, hiring “crowd-workers” for mini-jobs, employing people as independent contractors etc., challenge our understanding of stable work-relations as basis of social security and social identification.

Most management theories, based on an understanding of stable employee-employer relationship reflect a past ideal and are based on misleading assumptions about contemporary labor-relations. It can be shown that the importance of work as a main pattern of social recognition is a result of social upheavals starting with the industrialization at the beginning of the nineteenth century. This process was accompanied by new ideas about society and economy formulated by the economic thinkers of the classical period. Seen from this perspective, the strong identification with work and career as expression of one’s self-realization is the heritage of the social and the political ideas of enlightenment and the classical economic thinkers with their promises of increased wealth and opulence for all by means of economic development.

With the beginning of the classical period economists started to focus on human labor as a key-factor for a country’s economic development. In this vein, representatives of classical economy not only discussed technical aspects of labor, like the preconditions and the benefits of the division of labor, but also moral questions, like fair wages or social justice. It was the common conviction of most of the political economists of the eighteenth and nineteenth centuries that economic development leads to a state of society where an educated workforce will earn a decent living and profit from the overall increase of wealth by attaining some comfort and material security. Several “pessimists”, as Thomas Malthus (1999 [1798]), Ricardo (2004 [1817], chap. V) or Mill (1967 [1845]), objected to this position pointing to the growth of population as the most pressing problem hindering the increase of workers’ remuneration. Nevertheless, even they admitted that a moderation in the reproduction rate of the working class by means of governmental social policy would stabilize the wage level at a decent minimum.

Also socialist critics of the capitalist system, like Owen (1840), Fourier (1841) or Louis Blanc (1848), did not follow the optimistic view of the representatives of classical economy and opposed their theories with a utopian idea of workers’ cooperatives and models of worker participation to overcome the systematic injustices of the capitalist system. While socialists remained modest concerning the reforms they proposed, Marxists suggested that the systematic failures of the capitalist system cannot be solved by social reforms but can be overcome only by a revolution of the working class abolishing the system as a whole (Marx and Engels 1848).

However, the prediction that economic theory would “place wages and laborers in fierce and eternal conflict with profits and capitalists” (Robinson 1826, 513) did not hold true in history. In opposition to David Ricardo’s and Karl Marx’s pessimistic view, that the use of machinery will throw laborers out of employment (Ricardo 2004, 390), and cause the crisis of capitalism due to the impoverishment of the working class (Marx 1974, III, 260), historical development took a different course. In fact, the increased productivity in the industrial production did also benefit the working class. As Andrew Ure already at the times of Marx could show with empirical data from the cotton industry, the improvement of the machinery did not only lead to an increased productivity in this sector but also to an increase of the wage level of the worker (Ure 1835, 319–321). Ure concludes that the “whole benefit arising from the improvement is divided between the master and the operative” (Ure 1835, 321).

Furthermore, by end of the nineteenth century unionization and diverse governmental regulations in industrialized countries led to improved working conditions by limiting working hours, enacting first factory security regulations and introducing social insurance systems. With beginning of the twentieth century social insurance and welfare systems and the enforcement of labor rights like collective bargaining or unionization moderated the former exploitative working conditions of industrial workers in the first-wave industrialized countries.

Although two world wars interrupted the progress of ameliorating the working-conditions of the laboring class, latest with the economic recovery after WWII the working class reached a status of affluence. In the following years, lifelong regular employment at decent wages, including social benefits and pension programs of the industrial workforce, became the default case for most of US-American and Western-European employees. It seems that the so-called “Social Question” has been settled and the promises of the “capitalist program”—“wealth for all” (Erhard 1957)—two hundred years after their first formulation have been fulfilled.

However, along with the advent of regular employment as standard case, employment not only became the precondition for social security in most Western economies, but also a prerequisite for social acceptance. Thus, “being unemployed” became a social stigma in a society where social participation and social security systems were based on lifetime employment. While the twentieth century system of work-relations was designed mainly for “blue collar workers” and was embedded in the context of national legislation, guaranteeing labor rights and providing social security and social insurance systems for the domestic workforce, work relations have changed by the turn of the millennium.

It is the aim of this contribution to show how our positive attitudes towards work, established by the political economic theories of the eighteenth and nineteenth centuries, are jeopardized by the social changes in post-industrialized societies. To do this the remaining part of this contribution is structured as follows. In a first step, we will show how the assumptions of classical economy formed a new idea of the laboring class as the backbone of the upcoming industrial society and depict the social consequences of this new view on labor. In a second step we will outline the challenges such laboring societies face when classical labor relations are endangered by increased unemployment and transformation processes in the industrial relations. Based on this we will analyze some new tendencies in the labor relations and discuss their implications in the next step. Finally, we will summarize our findings in the concluding chapter and show some implications for managerial theories based on such “old ideal” of labor relations.

## The Promises of the Classical Economy – and Its Social Consequences

Classical economy has been a typical child of enlightenment. In this vein, main convictions of enlightened philosophy, like rationality, liberty and autonomy as cornerstones of enlightened thinking, have been transferred to economic conceptions and have been re-labeled as economic liberalism, economic rationality and strong methodological individualism as premises of economic thinking. Adam Smith’s idea of “natural liberty” shaped an economic understanding where the individual knows best about his purposes and his capabilities to reach them. He, therefore, should be “left perfectly free to pursue his own interest his own way, and to bring both his industry and capital into competition with those of any other man, or order of man” (Smith 1981 [1776], IV.ix.51). In this line of thought, industry and individual efforts became basic preconditions for achieving prosperity and attaining a decent living. Adam Smith believes that a liberal economic system is a means of “liberating the poor from desperate need” (Schmidtz 2016, 208) and helps to increase the standard of material comfort of the laboring class (Winch 1978, 87). However, Smith is aware that such opulence is not shared equally, and he is “deeply concerned about the inequality and poverty that might survive in an otherwise successful market economy” (Sen 2016, 287–288). Thus Smith admits that the “division of opulence is not according to the work. The opulence of the merchant is greater than that of all his clerks, tho’ he works less; and they again have six times more than an equal number of artisans, who are more employed. The artisan who works at his ease within doors has far more than the poor laborer who trudges up and down without intermission. Thus he who, as it were, bears the burthen of society has the fewest advantages” (Smith 1982, 213). Nevertheless, Smith remains optimistic concerning the long-term effects of growing opulence and believed that also the working poor would profit from the overall economic development (Smith 1982, 211–212), and that attaining some comfort would also raise their moral and educational level (Smith 1981 [1776], I.viii.43; 1982, vi.6–7). On the long run, thus the belief of Smith, economic development would also lead to social justice and grant the laborers a fair share of the national product since “[n]o society surely be flourishing and happy, of which the far greater part of the members are poor and miserable. It is but equity, besides, that they who feed, clothe and lodge the whole body of the people, should have such a share of the produce of their own labor as to be themselves tolerably well fed, clothed and lodged” (Smith 1981 [1776], I.viii.36).

However, not all classical economists shared this optimistic view about the economic and social development based on the system of natural liberty. Starting with Joseph Townsend’s pamphlet “Dissertation on the Poor laws. By a Well-Wisher to Mankind” (1971 [1786]) a discussion about the “moral” conditions of the laboring poor has been initiated. By and large, the argument brought forward by Townsend was that lacking morality of the poor will doom them to a life in poverty and misery. Also other pessimists like Thomas Malthus, David Ricardo or John Stuart Mill identified lacking discipline of the laboring class with regard to their own reproduction as the key problem for stabilizing wages at a level that would allow laborers a decent living (Malthus 1999 [1798]; Mill 1965 [1848], Book II, chap. XI-XII). In this vein, governmental social programs like the British poor laws have only negative effects and, as David Ricardo criticizes, “instead of making the poor rich, they are calculated to make the rich poor” (Ricardo 2004 [1817], 96). Since the poor laws are rather based on a philanthropic ideal than on realistic assumptions, they are inappropriate to halt the unrestraint growth in population of the laboring class. Although several authors raised concerns about the validity of such an “iron law of population” (e.g. Whewell 1831, 160–168) as the main cause to depress wages to the subsistence minimum (e.g. Longe 1866; Thornton 1869), the growth in population became the main point in the discussion of how to remedy the social misery of the poor. To prevent the descent of wages to the level of starvation the only way would be to change the habits of the working poor regarding their reproduction rate by means of education and instruction, followed by gradually granting rights of political participation and, thus, creating a class of independent and self-reliant people (Ekelund and Tollison 1976).

However, the ideas how the laboring poor should be educated to become useful members in the new commercial society varied according to different authors. Especially the earliest writers, like John Locke (1997 [1697]) or Bernard Mandeville (1988 [1724]), recommended sometimes radical—and in the eyes of the today’s beholder—inhumane means to accustom the poor to labor. To teach them second order virtues, like temperance, punctuality, cleanliness or industriousness, they recommended to send the poor to working schools and houses of correction and to force them to labor by corporal punishments. The means chosen by the authors of the nineteenth century sound comparatively tame. Beside schooling and moral education, it is the central idea that workers should join in workers’ cooperatives where they take on the responsibilities for their own affairs. Such increased independence would create moral awareness and a sense of virtue on its own accord since workers’ cooperatives will function as „a course of education in those moral and active qualities by which alone success can be either deserved or attained “ (Mill 1965 [1848], 793). In his chapter “On the Probable Futurity of the Labouring Classes” in his “Principles of Political Economy” Mill develops a picture of fair and peaceful competition between capitalist enterprises and workers’ cooperatives organized by the workers themselves according to democratic standards and with fair shares of profit for each member (Mill 1965 [1848], Book IV, chap. VII). Notwithstanding the differences concerning the answer to the question of how the living standards of the working poor could be elevated, it was the common conviction of most political economists that a reasonably educated workforce would be able to find a decent living for them and their families. Changing the habits of the working poor and teaching them second-order virtues like punctuality, temperance, cleanliness, or industriousness would make them better citizens and enable them to reach a “state of affluence and some degree of reputation” (e.g. Franklin 1998, 3).

While modern economic theories focus especially on technical aspects like marginal productivity or marginal costs of labor to explain the wage level on the labor market and often ignore the social aspects in their economic theories, classical economists like John Stuart Mill saw economy also as a social and moral science (Mill 1965 [1848], 20f.). Seen from the perspective of the political economists of the eighteenth and nineteenth centuries, the later on so-called Social Question was social by nature and less an economic question. From their point of view, the knowledge of economic laws was useful as a precondition for viable social reforms (Hollander 1983), but economic laws could not determine the social goals of a society. For Mill the laws of the distribution of wealth in a given society are human institutions, “since the manner in which wealth is distributed in any given society, depends on the statutes or usages therein obtaining “ (Mill 1965 [1848], 21). For authors like Mill it was a matter of social justice that governments evolve the economic welfare of all citizens by enacting prudent social reforms.

Although the situation differed from country to country, the idea that social peace in the capitalist societies depends on the settlement of the Social Question and that prudent state reforms with regard to fair wages, employment security, social protection or workers’ rights will bridge “the fierce and eternal conflict” (Robinson 1826, 513) between laborers and capitalists became a common conviction. At the end of this process, a self-motivated and industrious workforce has become the backbone of the industrial society. At this point we can leave aside the question of whether this self-motivation to work was influenced by a protestant work ethics, as Weber (1993 [1904/5]) believed, or whether this was simply the result of the fully developed commercial society and the system of “natural liberty”, as it was the vision of Adam Smith. In the course of time, the self-motivation to work became a central part of the working ethos of the laboring class and was awarded by stable income and material security in the emerging industrial economy.

Following the ideas of the theorists—but also in reaction to the increasing pressure of unions and workers’ movements—with the beginning of the twentieth century, governments of nearly all industrialized countries enacted new legal regulations aiming at the emancipation of the working class by bestowing workers with rights of political participation and granting them co-determination rights at their workplaces. Simultaneously, workers profited from increases in productivity by raised wages and cheapened consumer goods. Although this development has been interrupted by two world wars and the economic crises of the 1920s and 1930s, the advancement of the working class did not halt in general. After WWII, the emancipated industrial workforce became the cornerstone of economic upswing and enabled Western countries to reach unprecedented economic success. At the same time, increased prosperity was spread across all layers in society, even, as Smith has predicted, not equally. However, in such work-centered societies “being employed” became the precondition for participating in the general upswing of the economic development. Thus, poverty has been replaced by unemployment as the new great threat of the working class in the industrialized countries, and it became the great challenge of governments to provide work for all by means of labor market policy and the establishment of social security systems for times of crisis. In a society where “being employed” is the ultimate proof of success and a precondition for social participation, unemployment endangers those affected to lose social status and to become ostracized from their former social community.

It was the promise of the Western economic system that education, individual efforts and disciplined work would lead to occupational advancement, foster individual career and increase personal material wealth, even for the lower classes in society. Consequently, labor has become the ultimate orientation of life in, as Hannah Arendt has called it, a laboring society (Arendt 1998, 4). In contemporary society, as Handy (2002, 26) has put it, we “seem to have made work into a god and then made it difficult for many to worship.” If every activity— from education over family to housekeeping—has to be labeled as work since this is the only valuable form of occupation, the absence of meaningful work is not only an economic but first of all a social problem endangering societal peace by disabling the unemployed to identify with central values of a work-based society. Hannah Arendt has addressed this in “The Human Condition” and critically asks the question what will happen in a society of laborers if people are freed from labor “and this society does no longer know of those other higher and more meaningful activities for the sake of which this freedom would deserve to be won” (Arendt 1998, 5).

## The New Challenges of the Laboring Society

With the upcoming of globalized economies, the digital transformation of industrial production and new industrial relations, like e.g. gig economy, part-time employment, outsourcing of services to independent contractors, posted workers, or personnel leasing, the conditions on the labor markets in Western economies have changed. Old patterns and promises of the industrial society seem to be outdated in a society where regular employment has become the exception. It is estimated that currently about 20 to 30 percent of the European and US-American workforce are hired as freelancers or “independent contractors” and another 10 percent involuntarily work in part time jobs, whereas the numbers are slightly higher in the US than in Europe (Kessler 2018, 9; Baldwin 2019, 141). From 1970 to 2017, full-time employment in manufacturing has dropped from 22 to 8 percent in the USA, from 23 to 9 percent in France and from 30 to 8 percent in the UK. Other industrialized countries face similar declines (Benanav 2020, 16). In line with this trend, the Organization for Economic Co-Operation and Development predicts that 14 percent of jobs in Western industrialized countries are at high risk to be eliminated during the next years due to automation and technical innovations in information technology, while another 32 percent will undergo fundamental changes (Benanav 2020, 5–6). Admittedly, these changes do not hit countries similarly. Depending on the characteristics of the industrial sectors and the respective workplaces dominating in different national economies, the potential of automation differs from country to country and even from region to region (Susskind 2021, 91–92). However, notwithstanding such differences in the regional developments, labor relations will change also in the less affected countries. Social security systems and workers’ participation mechanisms, developed over the past one hundred years in the industrialized Western countries, have been designed for labor relations based on full-time occupation and lifelong employment. In the “new economy”, such social security systems are no longer compatible with the structures of the newly developed labor relations.

Over the past three decades, mainly social scientists have analyzed the changing working conditions of the western laboring society, and have tried to outline various utopias of the new world of labor. Thus, for example, Juliet Schor (1993) describes how our “positive” attitude towards work generates an “overworked” society where even leisure has to be organized in a work-like manner and the “harried leisure class (…) would do more things at once and do them faster” (Schor 1993, 23). Based on statistical data, Schor shows that although in the United States industrial productivity has increased steadily after WWII, working hours have not been lessened. Instead of working—and earning—less, US-American workers today work more than their fellow workers did in the 1970s (Schor 1993, 2–5). On the other hand, due to the increased productivity, job-offers declined on the labor market, and due to the oversupply of labor hourly wages have fallen over the past decades. Consumerism and a constantly increasing living standard forces Americans to work harder and longer to attain desired goods, despite all increased productivity. “Capitalism”, thus the analysis of Juliet Schor, “has brought a dramatically increased standard of living, but at the cost of a much more demanding work life” (Schor 1993, 10). In this vein, also job quality has changed. Many Americans in low-paid jobs have to take second or third part-time jobs to finance consumer goods or pay off debts (Schor 1993, 31). For keeping up “with the ever-more-expensive middle- or upper-middle-class life style, more work has been necessary” (Schor 1993, 81).

However, in this “overworked society” labor relations have changed over time with not only economic but also social consequences. In this vein, for instance, Sennett (1998) shows how new working conditions form the character of a new working class in the US. Comparing work characteristics and work identifications of two generations Sennett analyzes the differences in their respective work relations. For the routine-workers of the mid-twentieth century, individual careers depended on loyalty and individual efforts in long-term and stable work relations allowing, at least to some extent, to plan one’s own “biography”. In a world with mainly task-group oriented jobs at different locations and with different partners and fellow-workers, this option has disappeared since there is no visible upward or downward development, but simply a sideward movement from one job to another without clear knowledge about whether this will be a step upward or downward on the career ladder. Such “drift” forms a new character, “since the experience of disjointed time threatening the ability of people to form their characters into sustained narratives” (Sennett 1998, 31). Such narratives in the eyes of Sennett are important as “they give shape to the forward movement of time” (Sennett 1998, 30) and give meaning to individual decisions in the sense that they are parts of people’s individual efforts to master their lives. In a more recent publication, Sennett shows how this changes in the work-relations lead to the erosion of former central values, which have shaped organizational culture, like respect, loyalty and solidarity (Sennett 2013, chap. 5).

Under such new conditions “new competencies” are required form the new “portfolio workers” in the future (Handy 1998, 26–27). In this vein, Charles Handy describes the trend that organizations increasingly will rely on self-employed knowledge-workers in the future. In this model, full-time jobs will be reduced to a minimum with the consequence that old security systems with pension plans and workers’ benefits will not work any longer. Under such new working conditions, the promise of lifelong employment becomes questionable. “It is bad economics because it puts the organization into a straitjacket and limits its flexibility. It is bad morals because it promises (…) what it cannot and will not deliver to more than a few” (Handy 1998, 89). Nevertheless, Handy remains optimistic. If it is a hallmark of the upcoming post-industrial society, that knowledge becomes the new production factor, then—in opposite to what Marx has believed—the workers remain the owners of the production means (Handy 2002, 23–24). But if “brain-work” becomes the major resource in a “knowledge-based economy” this also changes the structures of organization and of organizational hierarchies (Baldwin 2019, 141–143). Work can be done from a remote location or at home. Working hours become fluid and are no longer bound to company-equipment or specific working hours (Handy 2002, 215–217). In fact, the COVID-19 crisis can be seen as a lab to test such new labor relations with increased flexibility and new working routines. However, home offices also show that knowledge based work can easily been outsourced, not only to different workers but also to random places.

Additionally, increased advancements in information and communication technology will lead to automation also in the service sector, which hitherto has been seen as a stable source for employment. Quite optimistically, Jeremy Rifkin outlines the utopia of increased spare time due to increased productivity. Though he warns about the social consequences resulting from the “end of work” (2000) in the traditional understanding, he, nevertheless, sees a bright future of a “zero marginal cost society” (2015) which will allow people to use their spare time for voluntary activities in the third sector. Since knowledge is a free commodity, it can be shared with others, and since knowledge is the basis of future production, free access to knowledge will decrease marginal costs of production dramatically. “A big difference between digital technology and traditional technology is that new products and components can be reproduced costlessly, instantly and perfectly” (Baldwin 2019, 99). Having online free access to knowledge and education or to blueprints for goods allows people to get access to services like education nearly at no charge and to “produce” goods on their own 3D printer simply for material costs. Such innovations will change the economy and lead “into an era of nearly free goods and services” (Rifkin 2015, 5).

However, the future of the workers in this upcoming new economy looks less bright than Rifkin wants to make us believe. A question that Rifkin is not addressing concerns the problem of how wealth will be distributed in this new economy (Stiegler 2016, 179–180). Even if spare time is used for voluntary social engagement in the so-called “third sector”, such work has to be organized and depends on qualification. Since people still have to earn their living and social security is bound to the participation in the work process and regular work relations, the new “knowledge workers” and service providers cannot offer their services for free. Furthermore, they have to compete with a steadily increasing workforce in the knowledge and service sector and compete not only with their human competitors but also are threatened by the pressure of increasing automation. This puts a huge pressure on the wage level of the self-employed service and knowledge workers.

In the service sector, meanwhile new online platforms have created a basis for “borderless competition” even for well-educated academic freelancers, which now have to compete with suppliers from “low-cost countries” not less educated as they are, but offering their work at lower prices. Online platforms like “Upwork”, “Gigster”, “ZBJ” or “Mechanical Turk” make it easy to find freelancing knowledge workers for nearly every job that can be done on a computer while other sites like “TaskRabbit” or “Uber” offer service jobs and repair services or cab rides. Most workers in this “brave new world of labor” operate as independent contractors or freelancers, offering their labor at their own risk, and working beyond the regular social security systems and working conditions acts. Under most countries’ national law, they are not able to found unions, enjoy protection of anti-discrimination law guaranteeing equal treatment and equal pay, or have a right for collective bargaining. They have no regulated working hours and for most parts offer their work on demand.

## The Probable Futurity of the Working-Class

The explanations why our professional world is changing fundamentally in the coming years are multifaceted. Some authors see this as a consequence of saturated markets were growth of productivity exceeds the growth in sales of industrial products thus reducing the demand for labor in the manufacturing industries. The effect of this ongoing “deindustrialization” is twofold (Benanav 2020, 35–60): More and more workers from highly productive and, therefore, highly paid jobs in the manufacturing industry are moved to less productive and less paid jobs in the service economy, increasing competition in this sector (Susskind 2021, 109). Simultaneously, the overall decrease in economic development—caused by the slack in industrial production—reduces overall income and thus puts pressure on the wage level in the service sector. This might explain why “new ideas” fostering precarious working conditions for service workers in the so-called gig economy are so successful.

Other authors see the developments of the labor markets as a change in the service sector which formerly has been considered to be sheltered from international competition since services are local and personal and have only limited potential for rationalization (Baldwin 2019, 115–127). Globalization and advancements in the information and communication technology—Baldwin (2019) calls the combination of these two effects “globotics”—have changed this fundamentally. They have not only created an international workforce of freelancers competing over available jobs for brain-workers—like for instance in the publishing business where many publishers have outsourced the editing process to low-cost countries—but also extremely poorly paid mini jobs for so-called crowd workers at new computer platforms like Mechanical Turk and others. Additionally, service firms, as Uber and others, have found ways to treat their workers as “independent contractors”, which allows these firms to reduce their labor costs and to shift business risks to their workforce. Thus, Susskind (2021, 156) summarizes: “for the most part, economies around the world are becoming more prosperous but also more unequal. And the main culprit in this is technological progress.”

Currently we see three developments in the labor relations endangering not only commonly accepted social role models of how a successful life should be in a laboring society, but also challenge old management theories and our social security system, which are based on the idea of lifelong employment and a minimum level of job security.

### Telemigrants and the new Global Competition

“Unlike the old globalization—when foreign competition meant foreign goods—globotics globalization will involve foreign people who are bringing direct international competition on pays and perks into offices and workplaces” (Baldwin 2019, 200). “Telemigration” opens a new phase in the process of globalization, and this will change the work opportunities of white-collar workers in industrialized countries (Baldwin 2019, 2). In the last few years, former language barriers became less threatening for telemigrants due to automatic translation programs like “Google Translate”. This opens up new changes for the “remote intelligence” even from countries where English is not the first language. This new remote intelligence from developing countries works at less hourly wages, due to lower living costs, and is willing to work more hours to finish projects in time (Baldwin 2019, 116–119). Working in different time zones enables companies to work 24 h with telemigrants whereas working-hours are limited in the home country and overtime has to be paid (Baldwin 2019, 120). From perspective of Western employees, it seems that the old recipe for becoming successfully employed and making a career—investing in skills, education, training and experience—are outdated in the new economy with global competition and an increasing number of freelancers from abroad who easily can replace domestic workers. Life-long employment guaranteeing social security and being the precondition for a career pathway has become the exception in the world of the new on-demand workers. While remote intelligence and few high-skilled workers in Western economies may profit from these developments providing them with more work flexibility and—seen from perspective of the remote intelligence—greater income opportunities, the great number of crowd workers hired for ridiculous money at platforms like Mechanical Turk do not. Crowd workers neither are protected by labor laws or social security systems nor are they paid accordingly to finance their own health insurance or pension plans. They are the new working poor working under precarious working conditions without protection by governments.

### Posted workforce and the Return of Sweat-Shop Labor

Commonly, less productive labor in Western economies is paid better than in less developed economies, since less productive labor profits from the wage level of the high productive workforce in the manufacturing industry. This might explain why low-skilled workers like construction workers, delivery services, harvesters, waiters or cleaners are paid at a comparatively high level compared with their productivity. However, new labor regulations have started to threaten the job security in these sectors. Thus, for instance, in the European Union (EU), the posted workers act allows employers to send employees to other EU member states to carry out services on a temporary basis while staying employed in their respective home countries. This means that foreign workers working in one EU-country are employed by a firm located in another and are paid and insured according to the respective domestic standards of the country of origin where pay is less and obligations for social insurances are not always mandatory. This option has changed working conditions in several sectors dramatically since hiring posted workers can be used as a way to bypass national wage regulations. Pointing to the situation in the construction sector Caro et al. (2015, 1601) show how hiring posted workers, mainly from Eastern European countries, via foreign subcontractors “has become one formulation employers use to avoid labor regulation and employ low-wage migrants in precarious jobs.” Most posted workers are ignorant about the wage level, regular working hours, health and safety standards and other labor regulations in the countries where they operate and willing to accept lower wages and social security standards. Since they have only limited language skills and often are kept separately from their domestic co-workers, it is easy for employers to exploit them by paying wages below national minimum wages, save social security payments and force these workers to work overtime (Aßländer 2021). This new opportunity of hiring posted workers instead of local workers puts pressure on many economic sectors, especially in the richer Western European countries and forces also local workers to accept worse laboring conditions with regard to payment, job security and workers’ benefits. It also weakens the position of workers’ unions concerning collective bargaining and improving labor standards. Competing with cheap workers from abroad paid by lower standards will reduce the wage level especially for less-skilled domestic workers.

### Precarious Working Conditions in the Gig Economy

With the so-called gig economy a new level in the transformation process from formerly regular employment to unregulated labor relations has been reached. If workers are treated as seemingly independent contractors, this helps companies to circumvent state-regulations for employer-responsibilities. This mainly concerns mandatory workers’ benefits and social insurance services. The advantages for companies are obvious: independent contractors take no paid holidays, they never ask for wage rises, they do not cost in slack times, they do not cost when they are ill, and they do not cause social security payments (Kessler 2018, 8). It is obvious that the advantages of the employers are the disadvantages of their employees. The mantra of such job-offers is largely the same: people working for our company like their flexibility and independence (Kessler 2018, 59). However, the question is who profits from such increased flexibility. For most workers in the gig economy, “this ‘flexibility’ feels more like instability” (Susskind 2021, 110). When looking at the wages of “independent contracting” service workers in the USA, a deeper analysis shows that they are earning about ten percent less than their fully employed colleagues in the same service sector and are often not provided with a retirement savings plan and social benefits (Kessler 2018, 87). Furthermore, independent contractors have no restrictions concerning their working hours, break times, holidays or minimum leave. While on the one hand a company like Uber, treating their drivers as independent contractors, cannot regulate their working hours directly, it is able to regulate work assignments by paying incentives or threatening with “deactivation” of the drivers from their drivers’ list, on the other (Kessler 2018, 103–111). Since drivers, in the case of Uber, work exclusively for Uber, they have no other choice than to accept the “scheduled” working hours. While, on the one hand, “independent contractors” are treated as employees being not allowed to work for other companies, they bear the full financial risk of an independent freelancer on the other. Given the increasing competition in the service sectors, “Uber-like” models of providing personal services will flourish in the future but at the social price of a steadily growing number of precarious workers.

## Conclusion

It took about two hundred years to form a well-educated, self-motivated and disciplined workforce. It was the great promise of the classical economists and the capitalist system that the new industries will provide job opportunities for all and that the increased productivity will benefit the working classes too. By preaching work-related virtues like industriousness, punctuality, or cleanliness as precondition of success, classical economists helped to form a disciplined and self-motivated workforce. At the same time, the industrial system awarded this kind of workers’ discipline with life-long employment and steadily growing remuneration. In the course of the late 19^th^ and beginning 20th centuries, working conditions have been improved, workers’ rights have been enforced and social security systems have been implemented. Latest after WWII “being employed” became the default case in the laboring society.

Now the economic conditions have changed. “In the first phase of industrialization, textile work moved from the cottages to large mills. Now office work is moving from large offices to twenty-first-century equivalent of cottages” (Baldwin 2019, 143). As consequence of the first phase of industrialization, new management theories have been developed to organize labor in the new large production sites. Theories like Scientific Management or the Human Relations movement started to analyze human labor as key factor of productivity on an empirical base and focused on stable work relations in the industrial sector. Measures like job rotation or job enrichment tried to motivate workers and to increase job satisfaction for the benefit of the corporation. While these theories can be seen as reaction to the organizational challenges of industrialized production, new challenges rise with the upcoming of decentralized and asymmetric labor relations in the new platform economy. Despite the question of how to organize the management process in such new labor relations, the main challenges concern questions of social security and the guarantee of labor rights in the post-industrial economy. While the change of the former family-based social security systems required new ideas about how to install a safety net for migrant workers coming from their villages to the industrial conurbations, the new changes of the worker-employer relations from full-time employment to independent contractors creates similar challenges for the social security system. Though, we “still live in an age of Labour, as we have since the Industrial Revolution began” (Susskind 2021, 99), the digital transformation and the concomitant new industrial relations do not only reduce work time and alter work opportunities, but will change the way how work will be done. In a laboring society, this affects various aspects of human life. This concerns not only what Sennett has called the “sustained narrative” of successful life, but also concerns questions of labor organization, work-life-balance, social security or workers’ participation rights. This also affects rather philosophical questions about our understanding of the meaning and the meaningfulness of work. If “work is the center of our lives and influences who we are, what we value, how we think, and all that we do and become (Gini 2000, 181), what then, Al Gini asks rightly, “happens if work goes away” (ibid.).

While it has been the promise of the classical economy that increased productivity will benefit all layers in society, we see today that manual work with less options for increasing productivity either is outsourced to sweatshops often located in low-cost countries or delegated to posted workforce from outside the industrialized countries. While it has been the promise of classical economists that education and self-discipline will allow for individual careers and will open up the pathway for a better future of one’s own children, we see today that even academic education will not guarantee occupational advancement or job-security. While it has been the promise of classical economists that life-long occupation provides social security and participation in society, the mixed employment biographies of today endanger social recognition and access to social security systems, which are designed for life-long occupational biographies. In such a new laboring society, classical work-related values become meaningless since they are no longer awarded by the economic system.

Given the increasing number of underpaid workforce in the gig economy, society will face an increasing old-age poverty and rising costs in health care. In this vein, the solutions for changing the precarious working conditions in the gig economy, proposed by some observers, sound like the solutions in the mid-nineteenth century sweatshop debate: workers’ cooperatives and workers’ codetermination (Kessler 2018, 209–2010). However, experience from the fight against sweated labor shows that more is needed to end the new “sweatshops” of the gig economy than weak and fragile workers’ cooperatives (Aßländer 2021).

While conceptions of corporate social responsibility (CSR) stress good labor relations, workers’ participation, equal payment and health and safety programs as part of corporate responsibilities, which urge companies to grant and monitor compliance with such workers’ rights in the whole supply-chain, contemporary CSR-conceptions turn a blind eye on the recent labor market developments in the industrialized countries. Problems like old-age poverty, pay inequalities or insufficient health care insurances are rarely addressed as part of corporations’ social responsibilities, although these problems for the most part are “produced” by corporations. In a similar vein, stakeholder theories focus on employees as primary stakeholders and discuss several moral rights of employees and corresponding duties of employers resulting from such stakeholder-relation. However, no stakeholder theory lists the “unemployed” among the relevant stakeholder groups, even if they are affected by corporate policies of outsourcing, hiring posted workforce from abroad or moving jobs to online platforms. If theories about meaningful work describe such meaningfulness as “job satisfaction” or “calling” (e.g. Michaelson 2021) or as a “supportive relationship” or “enhancement of workers’ potential” in an employer-employee relationship (e.g. Tablan 2015) such theories have only limited validity in the context of gig economy or crowd work. Managerial theories emphasizing “good work relations” as a means to increase productivity and work performance are based on an ideal of workers’ loyalty and work-satisfaction that is no longer valid in a gig economy. Seen from this perspective, corporate responsibilities with regard to labor markets have to be discussed from a broader view. To deal with these new challenges the discussion about CSR or stakeholder-management should widen the scope of analysis and focus not only on employees but also on those not being employed or outsourced as “independent contractors” due to strategic decisions. Although business models like that of Uber are discussed critically in literature, there are no systematic analyses about the corporate social responsibilities for the consequences of corporations’ personnel policy with regard to its social consequences, e.g. unemployment or old-age poverty. Here much more work has to be done, both empirically and theoretically.

However, also governments should react to the new developments in the labor markets to avoid negative social consequences. What we need are new ideas of how to assess the value of work in our “laboring society”. This means that the economic idea that wages are solely determined by marginal productivity should be replaced by the idea of pay related to the “social value” of work. Even if it is difficult to define the social value of distinct services with “mathematical accuracy”, especially in the context of social work in the health care and nursing sector, such reassessment is overdue. Furthermore, social security systems should be adopted to the new models of labor and to mixed employment biographies. If life-long employment in stable employer-employee relations becomes more and more an exception, we need new models for health insurance and retirement programs, which grant access to basic social security also for the “independent contractors” of the gig economy and the invisible crowd workers working in their home offices for pays below the poverty line. In this context, stricter legal regulations should protect crowd workers, “independent contractors” and posted workers from exploitation, e.g. by allowing unions, defining applicable minimum wage regulations or creating platforms for collective bargaining. Finally, business models, which are based on sub-contracting and outsourcing of services, should be regulated and strictly monitored by state agencies to prevent employers’ circumvention strategies in relation to social security costs.

In fact, these new challenges mirror the central question discussed already by the “fathers” of political economy: How to promote social justice so as not to leave anyone destitute while simultaneously maintaining entrepreneurial spirit to invest and to take economic risks.

Reacting to the new developments in the labor-relations needs public awareness, political pressure and new legal regulations, and maybe a new, less labor-centered mindset relativizing the importance of work in our lives. If social security systems are designed for life-long employed workforce and life-long employability cannot be guaranteed any longer, our social security systems have to be adopted to the new situation. This will be a huge task. However, changing our mindset, after having been told for over two hundred years that only productive labor provides a bright future, is even more demanding.
